# Nonstandard power grid frequency statistics across continents

**DOI:** 10.1038/s41598-025-25334-4

**Published:** 2025-11-04

**Authors:** Xinyi Wen, Mehrnaz Anvari, Leonardo Rydin Gorjão, G. Cigdem Yalcin, Veit Hagenmeyer, Benjamin Schäfer

**Affiliations:** 1 Institute for Automation and Applied Informatics (IAI), Karlsruhe Institute of Technology (KIT), 76344 Karlsruhe, Germany; 2 Fraunhofer-Institute for Algorithms and Scientific Computing (SCAI), 53757 Sankt Augustin, Germany; 3 Department of Environmental Sciences, Faculty of Science, Open University of the Netherlands, 6419AT Heerlen, The Netherlands; 4 Department of Physics, Istanbul University, 34134 Istanbul, Turkey; 5 Faculty of Science and Technology, Norwegian University of Life Sciences, 1432 Ås, Norway

**Keywords:** Bimodal, Power grid frequency, Linear test, Correlation, SDE modeling, Hurst exponent, Energy science and technology, Energy grids and networks, Statistics

## Abstract

Power-grid frequency reflects the balance between electricity supply and demand in a power system. Measuring the frequency and its variations allows monitoring of the power balance in the system and, thus, frequency grid stability. Gaining insight into the characteristics of frequency variations and defining precise evaluation metrics for these variations enable better assessment of the performance of forecasts and synthetic models of the power-grid frequency. Previous work on the power grid frequency analysis was limited to a few geographical regions and did not quantify the observed effects. In the present contribution, we analyze and quantify the statistical and stochastic properties of self-recorded power-grid frequency data from various synchronous areas in Asia, Australia, and Europe at a sampling resolution of one second. Revealing non-standard statistics of both empirical and synthetic frequency data, we effectively constrain the space of possible (stochastic) power-grid frequency models and share a range of analysis tools to benchmark any model or characterize empirical data. Furthermore, we emphasize the need to analyze data from a large range of synchronous areas to obtain generally applicable models.

## Introduction

A power grid is a complex and interconnected network that enables the transmission and distribution of electricity from generators to consumers^1^. It is a vital infrastructure ensuring homes, companies, and industries have access to a robust supply of electricity^2^. To operate, a power grid must maintain a constant balance between electricity supply and demand. Any deviation from this balance can lead to grid instability, blackouts, or infrastructure damage. The generation portfolio’s diversity, integrating sources with different inertial contributions and ramp rates, directly influences this equilibrium and thus the system frequency. The operating voltage frequency of a power grid, denoted simply power-grid frequency, is a measurable quantity that indicates the operational status of a power grid. It reflects the rotational speed of the numerous synchronous machines within one area so that we refer to a region with one shared frequency as a synchronous area. An excessive feed-in of power into the grid causes an increase in frequency, whereas an insufficient supply results in a decrease in frequency. Sudden changes in this frequency can cause grid instability, which is why maintaining consistent frequency levels is paramount. The power-grid frequency typically remains within 1 percentage point of a reference value of 50 Hz or 60 Hz through the installation of various balancing and control systems in place^3^. These control systems monitor and stabilize the frequency to keep it within a permissible range^4–6^.

A thorough understanding and modeling of the power-grid frequency are necessary to use expensive control measures as efficiently as possible, e.g. via forecasting algorithms. Therefore, the analysis of the stochastic nature of the power-grid frequency has garnered substantial interest and attention from mathematicians, statisticians, and physicists alike^7,8^. Non-Gaussian frequency distributions have been discussed for European synchronous areas^9–11^, and frequency deviations have been successfully modeled using a Fokker–Planck approach^12^. However, a thorough characterization of the stochastic properties constraining potential models is missing. Moreover, previous works have often focused on European and African areas^9,13–15^, while for example measurements from Asia have not been thoroughly investigated or compared.

We substantially expand previous research^11,14^ by conducting a rigorous quantitative analysis of the statistical properties of a large class of synchronous areas. Our main objective is to establish quantitative measures that enable the comparison of different synchronous areas with each other and with synthetic models. To establish adequate models for power-grid frequency dynamics, we aim to understand how empirical data allows inferring of both the deterministic functions *g*(*f*, *t*) and stochastic contributions $\xi (f,t)$. Previous research assumed linearity in the deterministic part and Gaussian noise^16–18^. However, to enhance the adequacy of the model, we consider an equation of the form:

1 $$\begin{aligned} \frac{\textrm{d}f}{\textrm{d}t} = g(f, t) + \xi (f,t), \end{aligned}$$

where *f* is power-grid frequency, *g* is the (unknown) intrinsic dynamics of the system, and $\xi$ represents noise, e.g. $\xi =\frac{\textrm{d}W}{\textrm{d}t}$ where *W* could be a Wiener process. Both *g* and $\xi$ are potentially explicitly dependent on the frequency value *f* and time *t*. We now wish to understand how the empirical data constrains potential deterministic functions *g* or stochastic contributions $\xi$. Both *g*(*f*, *t*) and $\xi (f,t)$ can potentially take many forms, including potentially non-linear relationships in *g*(*f*, *t*) or correlations in $\xi (f,t)$.

This article is structured as follows: First, we give an overview of the multi-continent dataset. We then investigate fundamental statistical properties and the frequency distribution of empirical power grid data. Additionally, we compare the degree of bimodality across different synchronous areas. This comparison allows for an examination of how deterministic functions *g* influence the observed bimodal nature of the data. Next, we explore daytime versus nighttime dynamics to assess the potential influence of solar generation on frequency statistics. Following this, we extract empirical deadband ranges from the data by analyzing the first Kramers–Moyal coefficient, offering evidence that deadbands contribute to the observed bimodal frequency distributions. Furthermore, we calculate the one-step increment to evaluate frequency fluctuations. To establish a benchmark for comparison, we employ three distinct datasets derived from various stochastic differential models introduced in the reference^19^. We then evaluate and compare the degree of linearity in deterministic functions *g* between empirical power grid data and synthetic data. Lastly, we delve into a detailed analysis of the correlations in the system, suggesting long-range correlation behavior in the recorded frequency data. This analysis deepens our understanding of the behavior of stochastic contributions $\xi$. In the discussion and conclusion section, we present our findings and engage in a comprehensive discussion to provide a deeper understanding of the observed statistical properties and dynamics of the power-grid frequency.

## Data overview

Many previous studies, in particular ones discussing open data, have focused on European regions^12,13,20,21^. Meanwhile, there is some research^16,22^ that systematically and quantitatively compares the frequency characteristics of power grids across Asia, Australia, and Europe. One important reason is the limited availability of public data on power-grid frequency, making it difficult for researchers to comprehensively analyze the frequency behavior of power grids in different regions. To address this issue, it is necessary to encourage data sharing and collaboration among industry actors and academics—as well as to support initiatives that collect and disseminate such data. Furthermore, conducting comparative analyses that consider diverse geographical areas becomes essential. By undertaking a systematic study of power-grid frequency characteristics across different geographical areas we can examine the parallels, discrepancies, and underlying causes influencing power-grid frequency behavior.

To collect our dataset, we utilize a GPS-synchronized frequency acquisition device called an Electrical Data Recorder (EDR) developed at the Institute for Automation and Applied Informatics, Karlsruhe Institute of Technology, Germany^23,24^. The EDR provides data similar to a Phasor Measurement Unit while allowing easy transfer and processing of the raw and processed signals. Our primary mode of data collection involves connecting the EDR to conventional power sockets in an office or at a hotel to capture the voltage waveforms as experienced on the low-voltage distribution grid. Such local voltage phasor measurements allow the extraction of frequency values, which are essentially identical on the low-voltage and the high-voltage grid and thereby indicate the state of the entire synchronous area^20,25^. In the present study, we collected power-grid recordings from various locations in the Southeast Asian region, including Indonesia, Malaysia, and Singapore, and measurements from Australia.

**Fig. 1:**
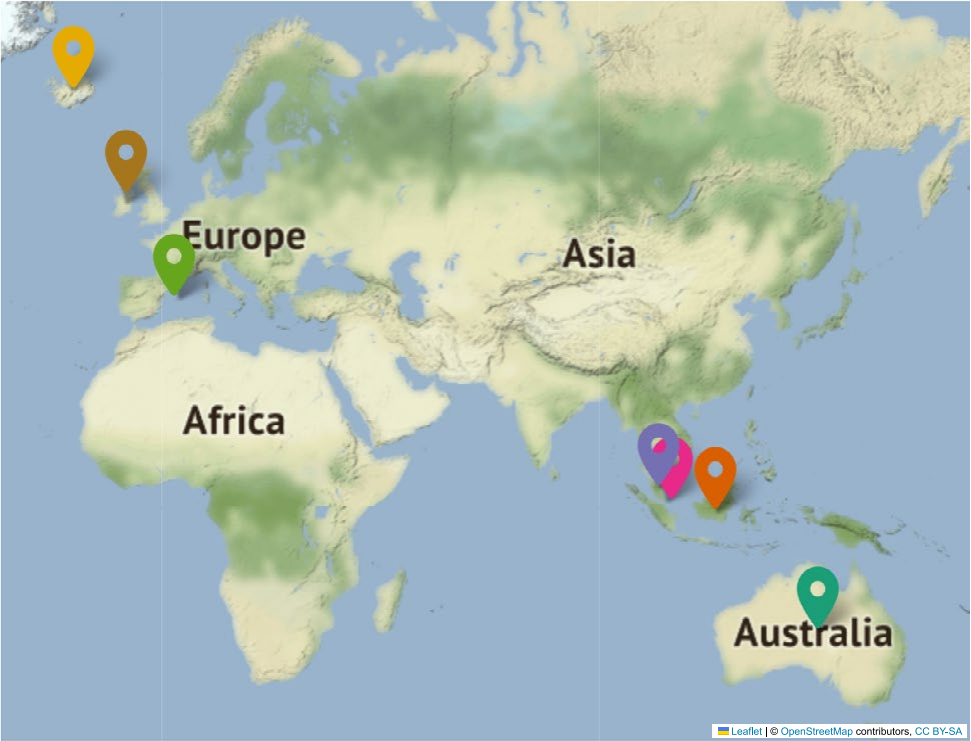
Location overview. Location of power-grid frequency recordings from Australia, Indonesia, Malaysia, Singapore, Iceland, Ireland, and the Balearic Islands. The map is created using the Folium package (Map data from OpenStreetMap).

The data collection period in Asia and Australia spanned 10–25 days at each location , from October 30th, 2022 to January 9th, 2023. We process the raw data to obtain frequency data with a resolution of one second. Furthermore, we also obtained power-grid frequency data from European areas, namely Iceland, Ireland, and the Balearic Islands. In European areas, where static measurement devices were employed, the data collection timeframe was longer compared to Asian countries, for which we utilized travel devices. In Europe. the data collection period ranges from September 29th, 2019 to February 22nd, 2022, covering at least three months in each location. In Fig. 1, we highlight the location of the measurement points on a world map. To perform an accurate analysis, we remove any intervals lacking EDR-recorded frequency data, ensuring a continuous dataset.

The selection of these countries in this study is based on their geographical diversity and distinct characteristics in terms of generation mixture and grid configurations and size. Specifically, we compare grids relying mostly on fossil fuels, such as Australia, where coal and natural gas together accounted for approximately 52% of total primary energy consumption in 2022–23, and fossil fuels overall made up 91% of the energy mix^26^, to those dominated by renewables such as wind energy and hydropower (e.g. Ireland) and sizes ranging from 370 thousand inhabitants (Iceland) to 280 million (Indonesia). This diversity in energy sources along with different operational control or market rules in each synchronous area contributes to variations in grid behavior and dynamics across these regions.

Additionally, we employ stochastic models, such as a Langevin process^19^ and a fractional Brownian motion-based model^27^ to generate synthetic data. Incorporating these synthetically generated datasets in our analysis serves a dual purpose: firstly, it allows us to gain a more comprehensive understanding of the underlying processes, and secondly, it enables us to validate and verify the efficacy of the methods we have employed in our study.

## Quantify bimodality

To gain an initial impression of the distribution of the power-grid frequency, we utilize kernel density estimation to display the probability density function (PDF). We compute the normalized third and fourth moment, i.e., the skewness and the kurtosis of the data. The skewness is a measure of the asymmetry of a distribution, with a value of zero indicating a symmetrical (potentially normally) distributed dataset. Meanwhile, kurtosis measures the behavior of the tails, i.e. of large deviations. A Gaussian distribution has a kurtosis of 3 so values below 3 indicate light tails and above 3 indicate heavy tails. We consistently observe kurtosis values below 3 in contrast to earlier results for European data^9,14^.

**Fig. 2:**
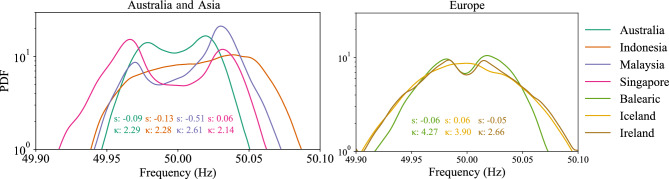
Frequency distribution. Most PDFs are shown on a vertical logarithmic scale. All power grids demonstrate non-Gaussian distributions, e.g. two peaks or non-Gaussian skewness *s* and kurtosis $\kappa$ ($s^\text {Gauss}=0$, $\kappa ^\text {Gauss}=3$).

All regions in our study operate at a reference value of 50 Hz around which the grid frequency fluctuates. Naively, one could expect that the most probable value of the grid frequency would be 50 Hz. Surprisingly, Fig. 2 shows that the PDF of the grid frequencies for various Asian regions exhibits a bimodal distribution, characterized by two peaks. This indicates that the power-grid frequency follows a bimodal distribution, rather than an unimodal distribution, potentially due to frequency deadbands in the control^12,19^. This bimodal behavior suggests that the grid frequency transitions between two discrete states, rather than varying continuously around the reference value.

**Fig. 3:**
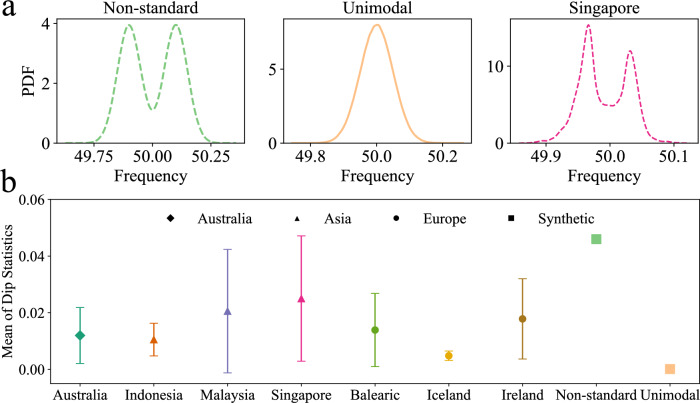
Bimodality quantification. **a** The left plot shows synthetic data with a bimodal distribution, while the middle plot displays a normal distribution derived from synthetic data as well. The right plot, based on actual data, reveals a bimodal distribution. **b** Using the mean value and standard deviation of dip statistics for hourly data, we measure the level of bimodality, with Singapore showing the highest value among the empirical datasets.

To illustrate the disparity in the distributional properties of our power-grid frequency data, Fig. 3a showcases three distinct density curves. The curves correspond to two synthetic baseline models and one empirical distribution observed in the data from Singapore.

To quantify the distributional properties of our power-grid frequency data, we calculate the dip statistic^28^, which measures the degree of bimodality, or equivalently, the deviation from unimodality. Specifically, it quantifies the distance between the empirical distribution and the closest unimodal distribution, with larger values indicating a greater departure from unimodality and providing more evidence for bimodality. To mitigate the impact of varying time frames on our analysis, we group the data into hourly intervals and then take the average dip statistic for blocks of 1-hour periods. Figure 3b provides an overview of the mean and standard deviation dip statistic values for the power-grid frequency data collected in our study, as well as the mean dip statistics of two synthetic datasets that follow a non-standard distribution and an unimodal distribution respectively, for reference. Singapore demonstrates the highest degree of bimodality among the datasets examined, as indicated by the largest value of the dip statistic. This result is consistent with our expectations, given the frequency distribution plot for Singapore shows the most pronounced double-peak pattern, see Fig. 2. Furthermore, our analysis indicates that Malaysia, the Balearic Islands, and Ireland also exhibit a bimodal distribution, evident from the relatively larger dip statistic values. Conversely, Iceland displays the smallest value of dip statistics. This aligns with our expectations because the PDF is more normally distributed compared to the other datasets^13^.

The observed bimodality indicates that the deterministic dynamics *g* in Eq. 1 could originate from a double-well potential or a superposition of single-well statistics (superstatistics)^9^.

## Impact of daytime

**Fig. 4:**
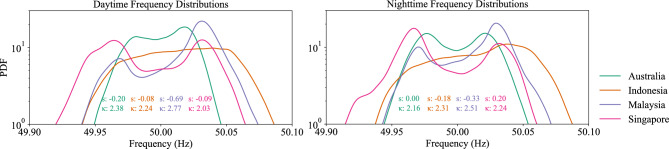
Daytime and nighttime frequency distribution. Probability density functions (PDFs) of grid frequency variations for Australia, Indonesia, Malaysia, and Singapore during daytime (left) and nighttime (right) periods. The distributions exhibit minor differences between day and night, with notable non-Gaussianity, non-stationarity, and bimodal features in some regions.

To explore the potential impact of solar generation on frequency dynamics^29^, we perform a basic analysis comparing frequency distributions during daytime and nighttime periods. Specifically, we divide the data into daytime (06:00–18:00) and nighttime (18:00–06:00) segments using local time for each region. Figure 4 presents the PDFs of the frequency distributions for Australia and Asian countries. Across all regions, we observe only minor differences between daytime and nighttime distributions. Both periods consistently exhibit non-Gaussian characteristics, and in several cases, bimodal structures are observed. This indicates that while day-time-specific generation (e.g. solar) or load may influence grid dynamics, its impact on the overall frequency distribution is likely small compared to other factors.

## Extracting empirical deadband ranges

**Fig. 5:**
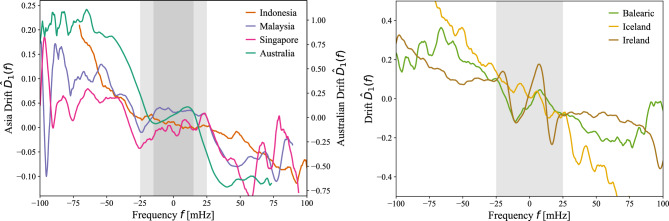
Drift Coefficient Analysis of Power-Grid Frequency. Estimated drift coefficient $\hat{D}_1(f)$ for Australia, Indonesia, Malaysia, Singapore (left) and Balearic, Iceland, Ireland (right). The linear response (negative slope) dominates the dynamics, with flat regions indicating frequency deadbands (dashed vertical lines and shaded areas). For reference, Australia operates with a deadband ±15 mHz^32^, Indonesia with of ±36 mHz^33^, Malaysia with ±25 mHz^34^, Ireland with ±15 mHz^35^, Iceland with deadband between ±10-30 mHz^36^.

To analyze whether frequency control deadbands contribute to the emergence of bimodal frequency distributions, we estimate the frequency deadband ranges directly from data using the first Kramers–Moyal coefficient, which captures the deterministic drift in frequency dynamics^30^. The drift captures the deterministic aspects of the dynamics in Eq. 1^31^, which we estimate as a time-independent function $\hat{D}_1(f)$. This is visible in the drift plots in Fig. 5, where the central plateau around 0 drift indicates the presence of a deadband, causing frequency to cluster at their edges and form bimodal distributions. We extracted the deadband values for Malaysia, Indonesia, and Singapore as ±25 mHz, and for Australia as ±15 mHz. For European countries, the deadband is also around ±25 mHz, while the data for Ireland appears to be noisy. These values agree approximately with the respective grid codes. Due to a lack of frequency control in these bands in the various power systems, this sometimes manifests as a positively sloped drift inside the frequency deadband, as can be seen for Singaporean data, and to some extent for the Balearic islands and Ireland, in Fig. 5. Observing a positive drift is counterintuitive as it implies the frequency is pushed *away* from its reference point of 50 Hz (60 Hz) and thus out of the deadband. This can happen due to longer-term control mechanisms, such as secondary and tertiary control. Empirical deadbands are estimated by finding the frequencies where the drift changes from zero or positive slope to a negative slope, which indicates control becomes active. Finally, we note that the (extracted and official) deadband values approximately coincide with the positions of the double peaks within the bimodal frequency distributions (Fig. 5). Hence, deadbands in the control scheme provide at least a partial explanation for this observation.

## Frequency increments

**Fig. 6:**
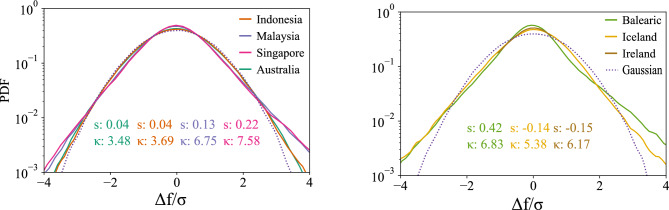
Distribution of increment frequency. Probability distributions of increment frequency on a vertical log scale. The variable *s* represents skewness, and $\kappa$ represents kurtosis.

To understand whether there exist sudden and extreme ramps or jumps in the frequency over time, we consider the distribution of the frequency increments for each region. For this purpose, first, we calculate the frequency increment $\Delta f_{\tau }=f(t+\tau )-f(t)$, where $\tau =1$ s, that is, the sampling rate of the data. We observe that the PDF of the frequency increments $\Delta f = \Delta f_{\tau =1}$ of each region, as shown in Fig. 6, exhibits deviations from a strict Gaussian distribution by displaying heavy tails for both negative and positive frequency increments.

Additionally, to provide insights into the statistical properties and distribution characteristics of the frequency increments, we calculate the skewness and kurtosis of the increments for each region. We observe that the Asian areas show positive skewness values, which can be understood as a ramp-up pattern, with a longer tail for positive values of the frequency increment distribution, see Fig. 6. On the contrary, Iceland and Ireland exhibit negative skewness values, indicating a leftward skew with a longer tail for negative values of the frequency increment distribution. Still, our analysis reveals that all regions display a very small non-zero skewness, indicating deviations from symmetry are minor, see Fig. 6.

Moreover, by measuring the kurtosis, we conclude that all synchronous areas exhibit a leptokurtic distribution with kurtosis values greater than 3. This suggests the presence of heavier tails in the frequency increment distribution compared to a Gaussian distribution. Our analysis indicates that the frequency increments in Asian, Australian, and European areas do not follow a Gaussian distribution, as evidenced by their leptokurtic distributions and non-standard statistical moments. These findings align with prior research^13,14^.

The observed heavy tails and deviations from Gaussianity indicate that the stochastic dynamics $\xi$ in Eq. 1 might be better modeled as a Lévy-stable process or a superposition of simple statistics than a simple Wiener process^9^.

## Linearity

In modeling power systems, there is often a preference for simplicity, and linear models are commonly employed. This simplification might contrast with the nature of power systems themselves, where, e.g. power flow equations are nonlinear by nature, but the inertial response from generating units is linear. However, it is unclear how this transfers to simplified models or empirical data of the power-grid frequency, and thus, we should test for the presence of linear and non-linear contributions in the data.

We use the higher-order autocorrelation function to test the linearity of the power-grid frequency data by quantifying the correlation between two observations in a time series at a given time lag^37^. The higher-order autocorrelation function of the frequency data provides a quantitative measure of time asymmetry in the dataset. If a time series exhibits asymmetry in time, it is indicative of non-linearity in the underlying dynamics. Therefore, we determine the degree of time asymmetry and quantify the level of non-linearity present in the power-grid frequency data. We calculate the higher-order autocorrelation for a given data set as:

2 $$\begin{aligned} LT (t) = \frac{[ f(t) - f(t + \tau ) ]^3}{[ f(t) - f(t + \tau ) ]^2}, \end{aligned}$$

where *LT* stands for “linear test”.

After computing *LT* for the original data, we also calculate *LT* on a surrogate time series to quantify the linearity. This involves taking the Fourier transform (*FT*) of the original data and randomizing the phases before using an inverse *FT* to obtain the surrogate data. The equation of Fourier transform (*FT*) is defined as:

3 $$\begin{aligned} F(\omega ) = \int \limits _{-\infty }^{\infty } f(t) \cdot e^{-i\omega t} \, dt, \end{aligned}$$

and its inverse is given by

4 $$\begin{aligned} f(t) = \frac{1}{2\pi } \int \limits _{-\infty }^{\infty } F(\omega ) \cdot e^{i\omega t} \, d\omega , \end{aligned}$$

where $\omega$ is the Fourier-frequency variable and $F(\omega )$ represents the Fourier transform of the function *f*(*t*) (the power-grid frequency in our case). The exponential term, $e^{-i\omega t}$, is the complex exponential function with an imaginary unit, *i*. The procedure described above, i.e., applying a Fourier transform, randomizing the phases, and applying the inverse Fourier transform, we effectively eliminate any non-linearity in the process, resulting in surrogate data that solely reflects the linear characteristics of the analyzed data^38^.

With the surrogate data at hand, we compute the *LT*(*t*) for the empirical and the surrogate data and then quantify the distance between both time series using the root mean square error (RMSE). If the value of the root mean square error (RMSE) is close to zero, it indicates that the time series exhibits linear behavior.

**Fig. 7:**
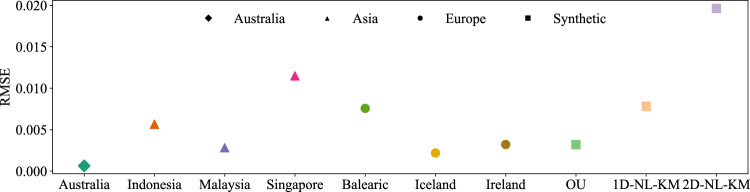
Linearity quantification. Visualizing the degree of linearity *LT* from Eq. 2, measured by the RMSE of data vs. surrogate data. Lower values of RMSE indicate a smaller deviation from linearity.

To validate our findings and put them into context, we employ three synthetic datasets of Ireland provided by stochastic differential models^19^. For the sake of brevity, we will not detail the models extensively and refer the reader to Oberhofer et al.^19^. We only include a short description of these stochastic models of the power-grid frequency, denoted as OU, 1D-NL-KM, and 2D-NL-KM, please check Appendix A for model equations. OU is a basic Ornstein–Uhlenbeck process with a single damping constant and noise term. 1D-NL-KM represents a one-dimensional non-linear Kramers–Moyal model that incorporates a non-linear response and multiplicative noise. 2D-NL-KM is a two-dimensional non-linear Kramers–Moyal model that separates the frequency into stochastic fluctuations and a deterministic trend. Hence, OU is fully linear, while 1D-NL-KM and 2D-NL-KM are designed to include non-linear effects. Our RMSE results attest to the nature of these models, as OU exhibits the lowest nonlinearity while 2D-NL-KM demonstrates the highest nonlinearity, as measured by their respective RMSE scores in Fig. 7. After analyzing the RMSE of the observed power-grid frequency data and three synthetic datasets, we find that the power-grid frequency data from Australia exhibits a linear property, characterized by small RMSE values of the *LT*(*t*) test. Meanwhile, the Singaporean and Balearic regions exhibit the greatest RMSE values, indicating a larger deviation from linearity, see Fig. 7. However, even the largest RMSE value for the power-grid frequency data is significantly smaller than the non-linearity observed in the synthetic models.

The observed linearity indicates that the deterministic dynamics *g* in Eq. 1 should approximately follow a linear relationship, which supports the idea of a superposition of simple statistics^9^ over explicit nonlinear modeling^12,19^.

## Correlation analysis

When modeling stochastic processes, it is key to assess whether long- or short-range correlations are present. Hence, we investigate the autocorrelation and decay characteristics of the datasets for Asian, Australian, and European power grids. The autocorrelation function (ACF) at lag $\tau$ of a time series $f_t$ is calculated as:

5 $$\begin{aligned} \text {ACF}(\tau ) = \frac{\text {Cov}(f_t, f_{t-\tau })}{\sqrt{\text {Var}(f_t) \cdot \text {Var}(f_{t-\tau })}}, \end{aligned}$$

where $\text {Cov}(f_t, f_{t-\tau })$ is the covariance between $f_t$ and $f_{t-\tau }$, $\text {Var}(f_t)$ is the variance of the original series at time $t$, and $\text {Var}(f_{t-\tau })$ is the variance of the lagged series at time $t-\tau$.

**Fig. 8:**
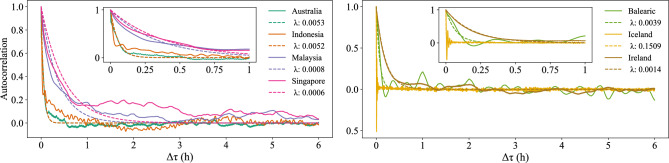
Decay of the autocorrelation. We calculate the autocorrelation for each region over a time lag of up to 6 hours. The solid lines represent the autocorrelation, while the dashed lines correspond to the exponential fit with a decay constant $\lambda$.

As shown in Fig. 8, the ACF of these regions’ power-grid frequencies exhibits an approximately exponential decay pattern concerning the time lag $\Delta \tau$. To quantify the decay trend, we fit a curve with $e^{-\lambda \Delta \tau }$ (where $\lambda$ represents the decay constant and $\Delta \tau$ is the time lag) using an exponential model. We observe that Iceland exhibits the highest decay constant value, measuring 0.1509, indicating a relatively rapid decay in autocorrelation. On the other hand, Singapore shows the lowest decay value at 0.0006, suggesting long-lasting correlations, potentially arising from correlated noise. The results are robust regardless of whether we consider 1 or 6 hours of data for the exponential fit.

**Fig. 9:**
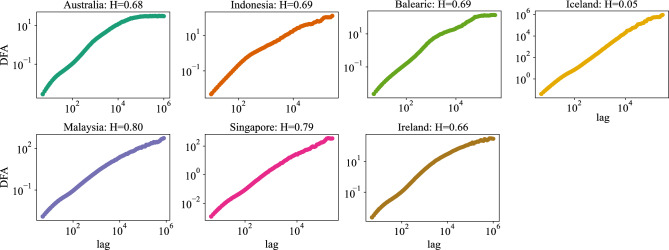
Computing Hurst exponents. We perform a Detrended Fluctuation Analysis. With the exception of Iceland, all regions exhibit Fluctuation Function patterns typified by Hurst values greater than 0.5.

To quantify these emerging correlations, we calculate the Hurst exponent of power-grid frequency for each region. Specifically, we estimate the Hurst exponent of these time series using the Detrended Fluctuation Analysis (DFA) method^39–41^. Several studies have successfully applied DFA to analyze power-grid frequency data and uncover underlying long-range correlations^21,42^. DFA stands out among other methods for its ability to accurately quantify the strength of long-range correlations, even when dealing with non-stationary time series^43^.

We generate a set of lag values that span from 5 to 10^6^. Figure 9 illustrates the DFA results of the power-grid frequency in various synchronous areas, utilizing the fluctuation function plotted against lag values. The slope of the fitted line in the log-log scale is equal to the Hurst index plus 1, in consideration of the integration performed in the DFA algorithm. From the slope of the Fluctuation Function, we extract the Hurst exponents, which exceed 0.5 for all regions except Iceland. This indicates the presence of positively correlated motions, i.e., long-range correlation.

These observed correlations indicate that the stochastic dynamics $\xi$ in Eq. 1 might be better modeled as colored or fractional noise instead of a simple white noise process. Meanwhile, the almost exponential decay of the autocorrelation supports simple stochastic models, such as Ornstein–Uhlenbeck processes or extensions thereof.

## Discussion and conclusion

In the present study, we have collected and analyzed non-standard characteristics of the power-grid frequency data from Asia, Australia, and Europe. In particular, we show clear deviations from Gaussianity of both the frequency and its increment statistics. The study also employs dip statistics to reveal varying degrees of bimodality across different synchronous areas and utilizes Detrended Fluctuation Analysis to uncover long-term correlations within the data. Our code to compare models and data quantitatively is available on Github^44^. We advance previous data analyses^13,14^ by including more non-European grids in our analysis. We compare the observed statistical results to reference systems and provide insights into the similarities and differences in power-grid dynamics across regions.

Naively, one could expect that a large number of random perturbations on the power grid leads to Gaussian distributions. Instead, we observe very clear non-Gaussian distributions: Frequency statistics are highly bimodal and the frequency jumps (increments) are heavy-tailed. While the exact nature and origin of these properties might vary between different synchronous areas, our deadband analysis hints that the bimodal distributions could arise from frequency deadbands in the control schemes^12,19^ or transitions between two discrete states of the system. These transitions could be stochastic or deterministic, depending on the power system. Similarly, heavy tails in the increments are both plausibly explained as arising from sudden changes in power generation or load^10,14^ as well as by deterministic changes due to power dispatch at the start of an hour^45,46^. A further contributing factor to these non-Gaussian features may lie in the ongoing energy transition. The increasing share of inverter-based renewable energy sources, such as wind and solar, introduces fundamentally different dynamics to power grids. These resources lack the physical inertia provided by conventional synchronous generators^47^. This reduction in system-wide inertia leads to larger and more rapid frequency deviations in response to supply-demand imbalances. Demand-side flexibility, particularly demand response, is becoming an increasingly important factor influencing frequency statistics. Demand response can operate on fast timescales–up to 4 seconds–allowing for rapid response to frequency deviations. As such, the observed heavy tails and, in some regions, bimodal distributions in frequency statistics may reflect the statistical features of low-inertia operation^48^.

As with Gaussianity, a common assumption about stochastic processes is to regard them as uncorrelated. Again, we found that power grids both in Asia and Europe are more complex than this simple assumption. Furthermore, utilizing Detrended Fluctuation Analysis (DFA), we demonstrated that the Hurst values generally range from 0.7 to 0.8, i.e. the time series displays a positive self-correlation. This finding is consistent with previous studies in this field^49^. Power grids still consist of a large number of synchronous generators thereby rotating mass with inertia. This already makes more continuous and correlated dynamics plausible. In addition, fluctuations both from the consumer^50^ and from the generation side will often be correlated^10^. Meanwhile, the full data displayed mostly linear properties, potentially simplifying at least one modeling aspect.

Let us review the added benefit of considering measurements from diverse geographic regions, instead of limiting ourselves to synchronous areas from one continent. While Singapore displayed a pronounced bimodality, there is no clear trend that Asian synchronous areas are more bimodal than European ones. Synchronous areas in Asia, Australia, and Europe all displayed heavy tails and mostly linear dynamics, with the two most non-linear areas (Balearic and Singapore) from different geographic regions. The correlation results differed the most: The Asian data sets returned a slightly higher Hurst exponent, while Iceland is the only anti-correlated area in our data set. Overall, we conclude that we require many different synchronous areas to have access to interesting dynamics as in Singapore or Iceland. Including data from multiple geographic regions will likely increase the chance of observing non-standard effects that have to be incorporated into any general-purpose model.

Concluding, our findings advance our understanding of power grids and their simulations. Regardless of the origin of the added complexity (bimodal, non-Gaussian fluctuations, long-range correlations), power grid models, such as the simplified Eq. 1 should take these deviations into account and benchmark their models against empirical data, whether they are using linear^51–53^ or non-linear^12,19^ models to be applicable to as many different settings as possible.

In the future, there are several other prospective studies and analytical options to continue the work presented here. One intriguing area of research is the comparison of frequency dynamics between different seasons, particularly winter and summer. Seasonal variations in power demand and generation patterns can significantly influence frequency behavior, and exploring these variations could provide valuable insights into the system’s response to changing operational conditions. In addition, examining power-grid frequency dynamics at different resolutions, such as milliseconds, hourly, or daily, can provide a more comprehensive understanding of the underlying dynamics and fluctuations. While we provide some initial analysis of how daytime affects frequency statistics, a thorough and systematic study, e.g. superstatistical effects^9^, is left for future work. Furthermore, investigating the interdependencies and interactions between power-grid frequency and other critical system variables, such as voltage amplitudes and power flows, could be beneficial to gain a more comprehensive understanding of the system’s behavior. Another important direction is to study the impact of large-scale Battery Energy Storage Systems (BESS) on frequency dynamics. BESS are increasingly deployed to provide fast frequency response and synthetic inertia, which inherently can alter frequency statistics. A more detailed investigation, interconnecting the action of BESS on grid frequency, is in order, where data is available.

Finally, our examination can be extended to islanded and microgrids operated primarily using power electronics to assess if different statistical and stochastic properties are present.

## Data Availability

Frequency data are available openly on https://power-grid-frequency.org/^54^ or upon reasonable request from the corresponding author.

## Appendix A: Models for generating synthetic dataset

We use three different models to generate synthetic datasets, denoted as OU, 1D-NL-KM, and 2D-NL-KM.

- **OU Model:**
  $$\begin{aligned} \dot{\theta }&= \omega \\ \dot{\omega }&= c_1 \omega + \epsilon \xi \end{aligned}$$
- **1D-NL-KM Model:**
  $$\begin{aligned} \dot{\theta }&= \omega \\ \dot{\omega }&= c_1(\omega ) + c_2(\theta , \omega ) + \Delta P + \epsilon (\omega ) \xi \end{aligned}$$
- **2D-NL-KM Model:**
  $$\begin{aligned} \dot{\theta }&= \omega _{\text {fluct}} \\ \dot{\omega }&= c_1 (\omega _{\text {fluct}}) + c_2 (\theta _{\text {fluct}}) + \Delta P + \epsilon (\omega _{\text {fluct}}) \xi \\ \omega&= \omega _{\text {fluct}} + \omega _{\text {trend}} \end{aligned}$$

Where $\theta$ is the bulk angle of the voltage, $\omega$ represents bulk angular velocity, $\omega = 2\pi (f - f_{\text {ref}})$, with the bulk frequency $f$ and reference frequency $f_{\text {ref}} = 50$ Hz. $\Delta P$ is the power mismatch. The symbols $\epsilon$ and $\xi$ represent the noise amplitude and Gaussian white noise function, respectively. $c_1$ and $c_2$ are control parameters estimated from the Kramers–Moyal coefficients. The term $c_1(\omega )$ models the combination of primary control $c_1$ as well as the proportional part of secondary control $c_2$, and $c_2(\theta )$ models the integral component of secondary control $c_2$. Detailed properties and modeling methods are extensively explained in the references^16,19^.
